# High-Grade Serous Ovarian Cancer during Pregnancy: From Diagnosis to Treatment

**DOI:** 10.3390/curroncol31040144

**Published:** 2024-04-02

**Authors:** Gregor Vivod, Sebastjan Merlo, Nina Kovacevic

**Affiliations:** 1 Department of Gynecological Oncology, Institute of Oncology Ljubljana, 1000 Ljubljana, Slovenia; gvivod@onko-i.si (G.V.); smerlo@onko-i.si (S.M.); 2 Faculty of Medicine, University of Ljubljana, 1000 Ljubljana, Slovenia; 3 Faculty of Health Care Angela Boskin, 4270 Jesenice, Slovenia

**Keywords:** ovarian cancer, cancer, high-grade serous ovarian cancer, pregnancy, surgery, chemotherapy, fetal outcome

## Abstract

Background: Due to the rarity of ovarian cancer diagnosed during pregnancy, the literature on the treatment of subtypes of epithelial ovarian cancer in pregnancy is sparse. The aim of our review was to analyze cases of high-grade serous ovarian cancer in pregnancy. Methods: The PubMed and Scopus databases were searched for relevant articles published in English between January 2000 and December 2023. The references of all the relevant reviews found were also checked to avoid omitting eligible studies. Information on the all retrieved cases was extracted and reviewed in detail. The most important detail was the subtype of high-grade serous ovarian cancer, which was referred to as serous adenocarcinoma (grade 2 or grade 3) in older cases. Results: We found eleven cases with relevant details of high-grade serous ovarian cancer diagnosed in pregnancy. Despite the small number of cases we found, our study demonstrated the importance of an accurate initial vaginal ultrasound at the first examination in pregnancy and the safety of diagnostic surgery and chemotherapy in pregnancy. Conclusions: There have not been long-term follow-ups of patients’ oncologic and obstetric outcomes. As patients should be comprehensively informed, more detailed case reports or series with longer follow-up periods are needed.

## 1. Introduction

The incidence of ovarian cancer in pregnancy is 1:12,000–47,000 pregnancies [1]. This rate of incidence is expected to increase due to the delay in childbearing age and universal noninvasive prenatal testing [2]. In general, ovarian masses are found in up to 4% of all pregnancies [3]. The vast majority of these masses are pregnancy-related and spontaneously resolve within weeks or sometimes months in most cases. However, 3–6% of persistent ovarian masses are malignant [4]. Ovarian cancer is the fifth most common malignancy diagnosed during pregnancy and the second most common of all gynecologic cancers after cervical cancer [5]. Epithelial malignant and borderline tumors are the most common histologies found in pregnancy, while germ cell tumors are uncommon. The majority (80%) of malignant ovarian tumors during pregnancy are diagnosed at an early stage [6].

The diagnosis of ovarian cancer in pregnancy is usually an incidental finding in the first or second trimester, as ovarian cancer is generally asymptomatic [7]. Correctly determining an ovarian mass during pregnancy can be difficult due to the increasing size of the uterus and the morphological changes that can be caused by the pregnancy itself. Transvaginal ultrasound is the preferred imaging modality to assess and characterize ovarian masses during pregnancy [3]. The most common ovarian masses in pregnancy are functional cysts, resembling a follicular cyst or corpus luteum. The sonographic features suggestive of malignancy are the same as in non-pregnant women. A particular challenge with pregnancy-related ovarian masses is the decidualization of ovarian endometriomas due to hormonal changes during pregnancy. In this case, the sonographic features may be misinterpreted as malignant and close follow-up is required [6]. If the ultrasound examination is not sufficient to determine the nature of an ovarian mass, magnetic resonance imaging can be performed after the first trimester [8]. Computed tomography (CT) is often considered the third choice of imaging modality in pregnancy [3,9]. The use of positron emission tomography (PET) in pregnancy has rarely been reported in the literature and is associated with an estimated level of radiation exposure for the fetus [10,11].

Tumor markers are less reliable in pregnant patients than in nonpregnant patients [12]. However, if malignancy is strongly suspected or detected, tumor markers can be valuable. In general, the tumor marker CA-125 may be elevated in normal pregnancies, while CEA, inhibin B, antimuellerian hormone, and lactate dehydrogenase remain within normal limits [13,14]. Alphafetoprotein is mainly secreted by the trophoblast and therefore cannot be used as a tumor marker. Tumor markers should be assessed at 1 to 3 months post partum [12].

The treatment of ovarian cancer during pregnancy depends mainly on the stage and weeks of pregnancy. Although surgery is possible in all trimesters, it is preferably performed in the (early) second trimester, when the risk of miscarriage is lower [15]. Early-stage ovarian cancer should be accurately staged, including intraperitoneal and retroperitoneal staging. For FIGO stages IA to IIA, pelvic and para-aortic lymph node dissection is recommended [16]. In advanced ovarian cancer in FIGO stages III and IV, termination of the pregnancy should be considered if the diagnosis is made in the first half of the pregnancy. In patients who prefer to preserve the pregnancy, an ovarian biopsy or salpingo-oopherectomy should be performed, and platinum-based chemotherapy should be administered during the pregnancy. In these cases, cytoreductive surgery should be planned after delivery, as surgery to define residual disease during pregnancy is not possible [16].

Paclitaxel plus carboplatin is the standard chemotherapy regimen for epithelial ovarian cancer and the recommended treatment regimen in pregnancy [16]. Chemotherapy is contraindicated in the first trimester of pregnancy to avoid interference with organogenesis, as early exposure has been associated with a 10–20% risk of severe malformations [17]. Exposure in the second and third trimester has been associated with fetal growth restriction, premature birth, a lower birth weight, and stillbirths. Maternal risks are mostly similar to those for non-pregnant women and include myelosuppression, especially neutropenia and sepsis [18]. Moreover, neoadjuvant chemotherapy for advanced epithelial cancer may be the only option to preserve the pregnancy [7]. Targeted therapies and intraperitoneal chemotherapy are contraindicated during pregnancy [19].

To our knowledge, the literature on the treatment of subtypes of epithelial ovarian cancer in pregnancy is sparse. There are only small studies and single clinical cases for certain subtypes of epithelial ovarian cancer in pregnancy [20,21]. In the larger reviews that do exist, all subtypes of ovarian cancer are usually analyzed and discussed together [22,23,24,25,26]. With advances in surgical techniques and new medical treatment options, different subtypes of ovarian cancer, such as epithelial ovarian cancer with serous, mucinous, endometrioid, and clear cell subtypes; sex-cord ovarian cancer with granulosa cell tumors and Sertoli–Leydig cell tumors; and germ cell ovarian cancer with immature teratomas, dysgerminomas, yolk sac tumors, and embryonal cancer are treated differently and individually, especially during pregnancy [4,27]. The aim of our review was to analyze cases of high-grade serous ovarian cancer in pregnancy.

## 2. Materials and Methods

### 2.1. Search Strategy

The PubMed and Scopus databases were searched for relevant articles published in English between January 2000 and December 2023. Our search strategy included the following terms: (Carcinoma, Ovarian Epithelial [mesh] OR Epithelial Ovarian Cancer* [tiab] OR Epithelial Ovarian Carcinoma* [tiab] OR Ovarian Epithelial Cancer* [tiab] OR Ovarian Epithelial Carcinoma* [tiab] OR Ovarian Epithelial and Non-epithelial Malignant Tumor* [tiab] OR Epithelial Malignant Neoplasms [tiab]) AND (Pregnancy [mesh] OR Pregnancy [tiab] OR Pregnan* [tiab] OR Pregnancies [tiab] OR Pregnant Women [mesh] OR Pregnant Women [tiab] OR Pregnant Woman [tiab] OR Pregnancy Complications, Neoplastic [mesh] OR Neoplastic Pregnancy Complication* [tiab] OR Epithelial Ovarian Cancer in Pregnancy [tiab] OR Epithelial Ovarian Cancer and Pregnancy [tiab] OR Epithelial Ovarian Cancer During Pregnancy [tiab]) AND 1 January 2000:1 January 2024 [Date—Publication]. The references of all relevant reviews found were also examined to avoid omitting suitable studies. In addition, references to related articles were searched to identify studies that might meet the criteria. Each article was assessed by three independent reviewers (G.V., N.K., and S.M.), and disagreements were resolved together.

### 2.2. Inclusion Criteria

The inclusion criteria were as follows: Women with a diagnosis of primary high-grade serous ovarian cancer diagnosed during pregnancy; all published prospective and retrospective studies and case reports containing patient-relevant information; type of surgery; use of chemotherapy drugs during pregnancy; outcome of patient and baby; and the outcome of pregnancy. In the case of duplicates in the literature, the most recent and comprehensive articles were selected.

### 2.3. Exclusion Criteria

The articles were excluded for one of the following reasons: Articles not specifying the subtype of ovarian cancer; tumors that were diagnosed before or after pregnancy; articles with multiple cases in which variables were analyzed as a group and that did not include separate data for high-grade serous ovarian cancer; case reports of non-malignant lesions; pregnant women without ovarian cancer; or incomplete data.

### 2.4. Data Extraction

The following information was extracted and reviewed in detail: Patient age at diagnosis, gestational age at diagnosis, tumor location, histopathological subtype, International Federation of Gynecology and Obstetrics (FIGO) stage, symptoms and signs, tumor size, treatment during pregnancy, gestational age at delivery, method of delivery, baby’s weight at delivery, treatment after pregnancy, woman’s and baby’s outcome, and follow-up period.

## 3. Results

### 3.1. Our Case

We would like to add to the literature and share our data on a patient with high-grade serous ovarian cancer diagnosed during pregnancy. A 36-year-old woman was pregnant for the fourth time. In her obstetric history, the patient reported two spontaneous abortions and a successful pregnancy at the age of 27 years. She was healthy and did not receive regular treatment. Her family history revealed that one grandparent had colorectal cancer and two aunts had breast cancer. An early transvaginal ultrasound scan in the first trimester by her gynecologist did not reveal any adnexal masses. The pregnancy progressed normally until 21 weeks of gestation, when she presented for evaluation at the referring hospital because of persistent pelvic pain, lower extremity pain, and urinary urgency that had persisted for a week. The ultrasound scan revealed no abnormalities of fetal growth but showed a 94 mm × 64 mm ovarian mass on the left side, which was multiloculated and had no thickening of the septa. The right ovary was normal.

**Figure 1:**
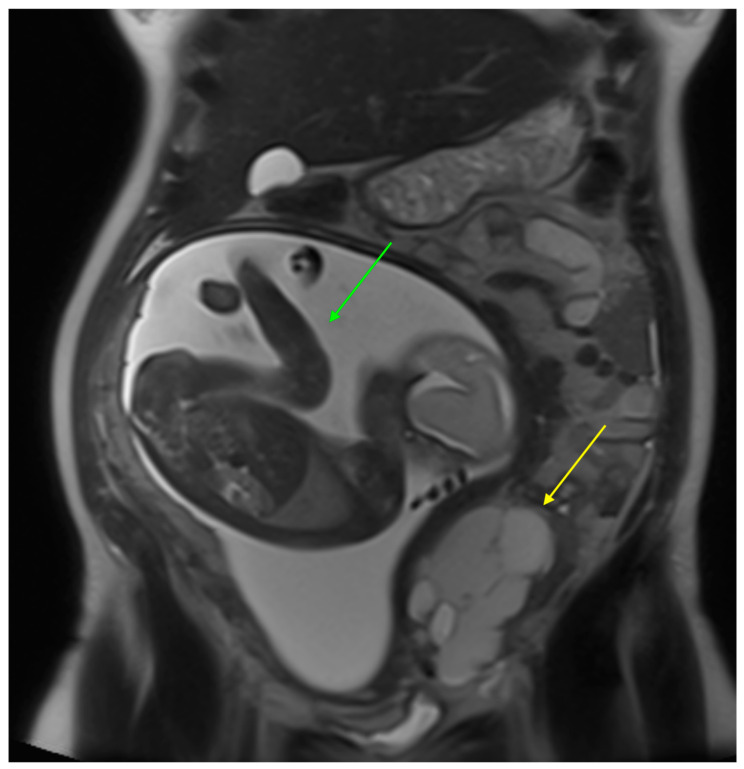
Abdominal MRI, sagittal view of the fetus (green arrow) and a high-grade serous ovarian cancer (yellow arrow).

**Figure 2:**
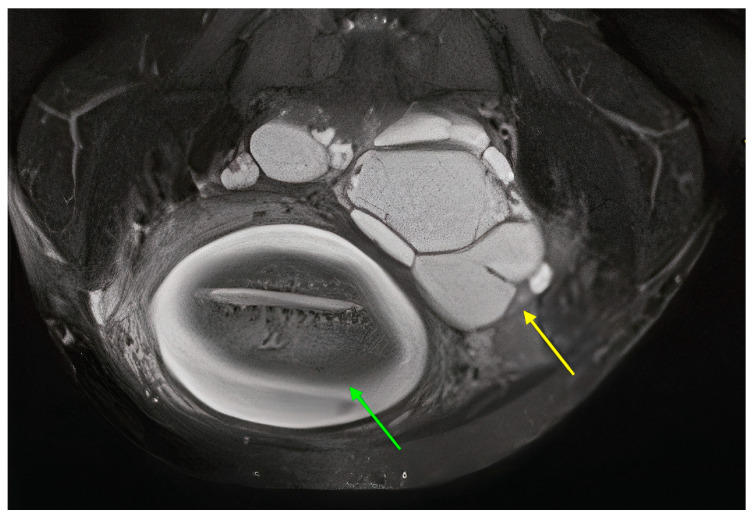
Abdominal MRI, transverse view of the fetus (green arrow) and a high-grade serous ovarian cancer (yellow arrow).

The follow-up ultrasound at the referring hospital two weeks later showed that both ovaries were multiloculated, the left ovary with a maximum diameter of 75 × 58 mm and the right ovary with a maximum diameter of 40 × 50 mm. There were no papillary protrusions, no solid areas, no blood flow, and no thickening of the septa and cyst walls. There was no ascites. Tumor marker testing revealed a Ca-125 level of 483 IU/L (normal range: <35 IU/mL) and an HE4 level of 179 pmol/L (normal range: <140 pmol/L). The patient was very motivated to continue with the pregnancy and continued to be monitored. At 26 weeks of gestation, the ultrasound examination revealed a multilocular right ovary with a maximum diameter of 80 × 100 mm, a multilocular left ovary with a maximum diameter of 120 × 100 mm, and echogenicity in one of the loci. In both ovaries, there were no solid areas, no blood flow, and no thickening of the septa. There was no ascites. A magnetic resonance imaging (MRI) scan of the pelvis and abdomen was performed and revealed a multilocular solid cystic tumor measuring 140 × 80 × 110 mm on the left ovary in conjunction with the right ovary (Figure 1 and Figure 2). Invasion of the rectosigmoid junction and vagina was suspected. The tumor extended to the anterior abdominal wall with suspected infiltration of the left rectus muscle. There was a 200 mm × 30 mm metastasis in the omentum. An 8 mm lymph node was suspected on the left side of the rectosigmoid junction.

A diagnostic laparoscopy was performed in the 28th week of pregnancy. It showed the presence of metastases up to 3 mm in size in the parietal peritoneum, metastases on both sides of the diaphragm, and carcinomatous omentum. Because of the gravid uterus, the lower part of the abdomen was not seen. Biopsies of the parietal peritoneum were taken and revealed a high-grade serous ovarian cancer at FIGO stage IIIC. A histologic analysis revealed that it was estrogen receptor-positive, progesterone receptor-positive, and positive for p53, WT1, p16, and Ki67. The following tumor markers were elevated: Ca-125 was 801 IU/L (normal range: <35 IU/mL), HE4 was 1457 pmol/L (normal range: <140 pmol/L), Ca 19-9 was 65 (normal range: <30 IU/mL), Ca 15-3 was 40 (normal range: <30 IU/mL), and alpha phetoprotein was 240 (normal range: <5.8 IU/mL).

The patient was presented to the interinstitutional tumor board, which included a gynecologic oncologist, a medical oncologist, a radiation oncologist, a radiologist, an obstetrician, and a pathologist. The interinstitutional tumor board decided to wait until the patient completed her 30th week of pregnancy and then to terminate the pregnancy by cesarean section and perform a cytoreductive surgery with intraperitoneal chemotherapy.

The patient was referred to our department. In her 31st week of pregnancy, a median laparotomy with a cesarean section was performed. The placenta was normal and showed no tumor infiltrates. The baby weighed 1360 g and had an Apgar score of 7 at one minute and 8 at five minutes. After the cesarean section, the presence of a multilocular ovarian cyst on the left side and a solid ovarian mass on the right side was confirmed. Carcinomatosis was present on the surface of the uterus, rectovaginal septum, rectosigmoid, cavum Douglas, both diaphragms, hepatoduodenal ligament, cecum, liver surface, mesentery, and both sides of the paracolic peritoneum. An omental cake was present. No ascitic fluid was found in the abdominal cavity during the procedure.

Subsequently, a cytoreductive surgery was performed, which included a hysterectomy with a bilateral salpingo-oophorectomy, total omentectomy, resection of the rectosigmoid colon with anastomosis, and pelvic peritonectomy. The metastases were coagulated on the liver surface and excised from the cecum, intestine, and mesentery. Macroscopic residual carcinomatosis of up to 10 mm was present on the diaphragmatic peritoneum, the mesentery, and the intestinal serosa. At the end of the operation, intraperitoneal chemotherapy with cisplatin was administered for 24 h.

The histopathological analyses confirmed a high-grade serous ovarian cancer. The right ovary measured 60 × 50 × 20 mm and the left ovary measured 120 × 90 × 50 mm, and both were overgrown with cancer cells. The fallopian tubes showed numerous superficial tumor infiltrates without invasion, and there were numerous metastases of high-grade serous ovarian cancer in the uterus, omentum, peritoneum, rectosigmoid, and cecum. The placenta was normal and showed no carcinomatous infiltrates.

After the delivery, the baby had to be intubated due to respiratory distress syndrome (the mother had received a standard dose of dexamethasone before the delivery). The baby’s respiratory function improved after the administration of a surfactant. The baby was extubated 7 days after the delivery and needed oxygen therapy until discharge. Due to the prematurity, bilateral retinopathy was present, which also improved with appropriate therapy. An ultrasound examination of the baby at 1 month of age showed a normal brain, heart, and kidneys. The baby was discharged home 2 months after birth, weighing 2760 g and requiring oxygen therapy. The oxygen therapy was gradually discontinued over the next few months. The child developed normally.

The patient recovered well after the operation and was discharged in good condition after 15 days. Four weeks after the surgery, the patient received the first cycle of standard adjuvant chemotherapy with paclitaxel and carboplatin for a total of six cycles 3 weeks apart. After the first cycle of chemotherapy, the tumor markers Ca-125 and HE4 were at a normal level. The patient was followed-up with regularly with ultrasound examinations of the abdomen and breasts as well as magnetic resonance imaging and a mammography of the breasts, all of which were negative. In addition, her clinical examinations and serum tumor markers (CA 125, HE4, CA 19-9, and CA 15-3) were within normal limits. The patient is currently without signs of disease 10 years after the diagnosis of high-grade serous ovarian cancer. The child, who is now 9 years old, has normal physical and neurological development.

### 3.2. Review of the Literature

**Figure 3:**
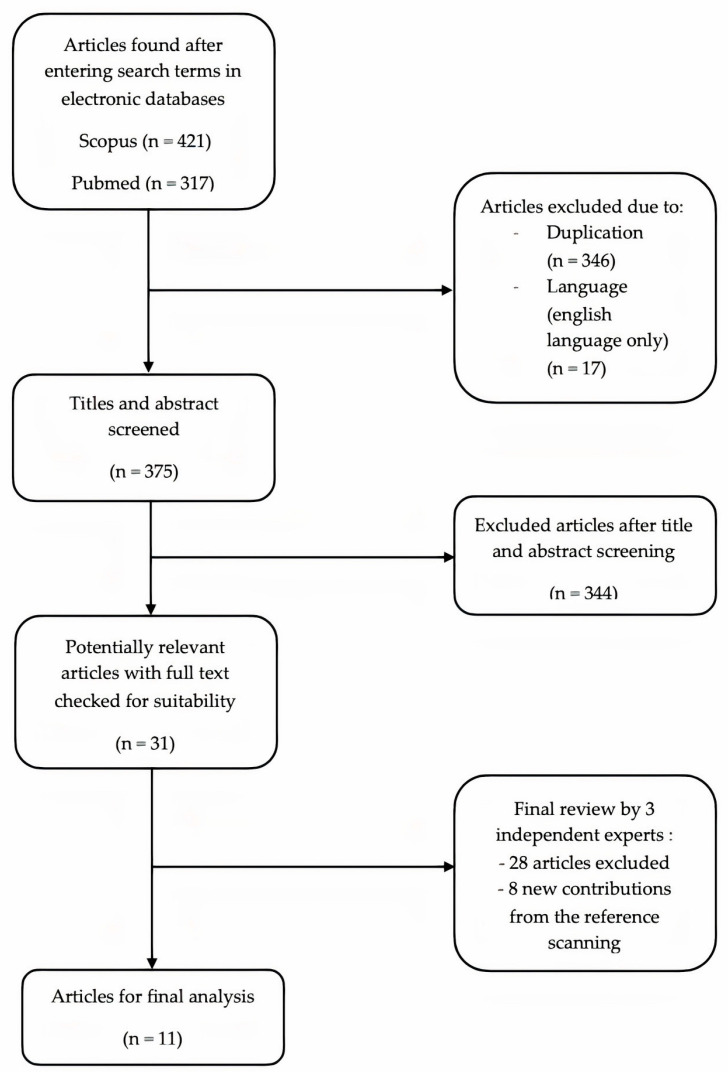
Literature review flowchart.

A flowchart showing the phases of our search strategy is shown in Figure 3. Our search in the Scopus and Pubmed databases initially returned 738 results. After the initial screening, 363 articles were excluded due to being duplicates or being written in a language other than English. Of the remaining 375 articles, the titles and abstracts were screened by reviewers, and 344 articles were excluded due to irrelevance. The remaining 31 papers that were classified as potentially relevant were subjected to a full text and literature review. In addition, 28 articles were excluded. Our evaluation of the references resulted in eight new relevant cases. For the final analysis, 11 cases with relevant details were selected [28,29,30,31,32,33,34,35,36,37,38]. The most important detail was the subtype of high-grade serous ovarian cancer, which was referred to as serous adenocarcinoma (grade 2 or grade 3) in older cases.

The mean age of the 11 women diagnosed with high-grade ovarian cancer during pregnancy was 33.9 years. Six women were asymptomatic at diagnosis and five women complained of pain. All the cases were initially diagnosed by ultrasound, and in three of the cases, magnetic resonance imaging was also performed. The mean gestational age at diagnosis was 14.9 weeks. Nine women were diagnosed at FIGO stage IIIC, one was diagnosed at FIGO stage IC, and one at FIGO stage IIB. The mean maximum diameter of the ovarian tumor was 103.7 mm. The mean value of the tumor marker Ca-125 was 584 U/mL at diagnosis. The detailed data of the patients and the tumor characteristics are listed in Table 1 and Table 2.

Ten women underwent surgeries during their pregnancies, nine women underwent exploratory laparotomies, and one woman underwent an exploratory laparoscopy. In one woman, her diagnosis was confirmed by an ultrasound-guided biopsy. The mean time of surgery during pregnancy was 17.5 weeks of gestation. Eight women underwent unilateral salpingo-oophorectomies. In all ten women who underwent surgeries during their pregnancies, the pregnancies were not affected. Nine women underwent cytoreductive surgeries at delivery, including cesarean sections and hysterectomies in all cases. One woman underwent HIPEC with cisplatin in addition to a cytoreductive surgery at delivery. In one case, the woman delivered vaginally at 37 weeks of gestation and underwent a complete cytoreductive surgery 6 weeks after delivery. In one woman, the pregnancy was terminated at 14 weeks of gestation and a complete cytoreductive surgery was performed immediately. Ten women received chemotherapy during pregnancy. Two women received cisplatin, two women received a combination of cisplatin and paclitaxel, one woman received carboplatin and paclitaxel, one woman received cisplatin and cyclophosphamide, two women received a combination of cisplatin and docetaxel, one woman received paclitaxel, and one woman received a combination of paclitaxel and intraperitoneal carboplatin. The details of the management of these cases of high-grade serous ovarian cancer during pregnancy are shown in Table 3.

The mean gestational age at delivery was 35.6 weeks. There were nine cesarean sections, one vaginal delivery, and one consensual abortion at 14 weeks of gestation. All ten pregnancies ended in a live birth. The mean weight of the babies at birth was 2512.6 g. Nine babies showed normal growth and development, with a mean follow-up time of 21.3 months (a range of 6 to 42 months). One baby died 5 days after birth due to multiple congenital anomalies which were diagnosed before the start of chemotherapy. Ten women received additional chemotherapy after pregnancy. Eight women were without signs of disease at a mean follow-up of 17.1 months (a range of 6 to 42 months) after delivery. One woman, who had a recurrence of ovarian cancer 24 months after delivery, was treated again with chemotherapy and was without signs of the disease 33 months after delivery. One woman died of recurrent ovarian cancer 29 months after diagnosis. For one case, there were no data on the woman’s management after delivery. The details of the patients’ obstetric outcomes and post-delivery management are shown in Table 4 and Table 5.

## 4. Discussion

To our knowledge, this is the first review to summarize all cases of high-grade serous ovarian cancer in pregnancy. Our review of the literature revealed that high-grade serous ovarian cancer in pregnancy is rare, and only 11 cases were described in detail. We would like to add our case and share our experience in the treatment of high-grade serous ovarian cancer diagnosed during pregnancy. Our case has the longest follow-up of ten years and showed successful treatment.

High-grade serous ovarian cancer is found at an advanced stage in more than 75% of cases in the general population [39,40,41]. In our case and in nine out of eleven cases diagnosed during pregnancy in our study, high-grade serous ovarian cancer was found at an advanced FIGO stage (IIIC). This underlines the importance of vaginal ultrasound at the first visit during pregnancy, when the ovaries should be scanned together with the uterus and the fetus. In cases of doubt and when additional information is required, magnetic resonance imaging is preferred as a non-ionizing imaging procedure to determine the size of the ovarian tumor, the extent of the invasion and lymph node involvement [16]. The use of gadolinium in magnetic resonance imaging is not recommended during pregnancy, as a recent study found that although gadolinium-enhanced magnetic resonance imaging at any gestational age was not associated with a higher risk of congenital anomalies, it was associated with an increased risk of a wide range of rheumatologic, inflammatory, or infiltrative skin diseases in the offspring as well as an increased risk of stillbirths or neonatal death [8]. Ionizing radiation techniques may only be used in individual cases after detailed discussions of the indication and clinical relevance and under strict and specific precautionary measures [16]. Although computed tomography can be used in pregnancy, its use could lead to fetal exposure to ionizing radiation and a theoretical risk of fetal thyroid suppression [3]. Reviews of the literature on the use of positron emission tomography in pregnancy are limited to case reports and small series and suggest general safety. To increase safety, a lower dose of 18F-FDG should be used in pregnant patients than in non-pregnant patients, and, if possible, PET should be performed alone instead of PET-CT. To facilitate the rapid elimination of the radiopharmaceutical, heavy hydration and a urinary catheter are recommended [11].

The tumor marker Ca-125 can be elevated during pregnancy up to a value of 200 U/mL [12,42,43]. The mean value of the tumor marker Ca-125 in our study was 584 U/mL at the time of diagnosis, which shows that the tumor marker Ca-125 is significantly elevated in cases of high-grade serous ovarian cancer.

General anesthesia is used for ovarian cancer surgery in pregnancy. The left lateral tilt position is recommended to avoid reduced blood flow to the placenta and fetal hypoxia due to maternal hypotension [16,44]. The risks of surgery in a pregnant patient include preterm delivery, miscarriage, and fetal distress. The physiological hemodynamic changes in pregnancy have implications for perioperative monitoring [16]. Careful preparation and the adequate monitoring of the maternal condition are essential for maternal and fetal well-being [44].

The mean time of surgery during pregnancy was 17.5 weeks, as it is recommended that surgery be performed in the second trimester [45,46,47]. For optimal exposure, a midline laparotomy is preferred, which is performed through a vertical incision in the midline with minimal manipulation of the uterus [7]. But the patients who underwent laparotomies for an ovarian mass during their pregnancies were significantly more likely to have preterm contractions than the women who underwent laparoscopies [48]. As with non-pregnant women, laparoscopies are associated with fewer fetal side effects, shorter operating times, and shorter hospital stays compared to laparotomies [49]. Laparoscopies are safe and feasible when specific guidelines are followed, such as the placement of the first troacar at least 3–4 cm above the uterine fundus, a maximum laparoscopic procedure time of 90 min, a pneumoperitoneum with a maximum intra-abdominal pressure of 10–13 mmHg, the use of the open introduction Hasson technique to avoid Veress needle injury, and performance of the procedure by an experienced laparoscopic surgeon [7,50]. In our study, nine women underwent exploratory laparotomies and one woman underwent an exploratory laparoscopy with no adverse effects on the fetus. In advanced-stage ovarian cancer (FIGO stages III and IV), cytoreduction to no residual disease is not possible during pregnancy. A biopsy or adnexectomy should be performed, followed by neoadjuvant chemotherapy with the completion of a cytoreductive surgery after delivery. Eight women underwent unilateral salpingo-oophorectomies and their pregnancies were not affected, confirming the safety of salpingo-oophorectomies during pregnancy [51].

Due to the low incidence of ovarian cancer during pregnancy and limited experience of this phenomenon, termination of pregnancy is usually reported in the literature [7,52]. In contrast, in our review, one woman decided to terminate her pregnancy in the 14th week of pregnancy, while ten women decided to continue their pregnancies. All of these women gave birth at a mean gestational age of 35.6 weeks with a mean baby weight of 2512.6 g. As recommended, nine women underwent cytoreductive surgeries at delivery and two women at 6 and 12 weeks after delivery.

Ten patients in our review received chemotherapy during pregnancy. The chemotherapeutic agents used were cisplatin, carboplatin, paclitaxel, and docetaxel, and in the oldest case from 1990, cyclophosphamide was used.

Since complete cytoreduction including hysterectomy is not possible during pregnancy, neoadjuvant chemotherapy might be suggested if advanced epithelial ovarian cancer is diagnosed during pregnancy [7]. The last cycle of chemotherapy should take place at least 3 weeks before birth to avoid hematopoietic suppression in the mother and myelosuppression in the newborn [53]. The standard chemotherapy treatment for high-grade serous ovarian cancer is paclitaxel plus platinum derivatives and is recommended in the second and third trimester of pregnancy [7]. Due to the risk of fetal malformations, the administration of platinum derivatives in the first trimester is not recommended [7,16,54]. Both cisplatin and carboplatin have been shown to cross the placental barrier, as both can be measured in the amniotic fluid and umbilical cords of the newborn [7,55]. No adverse effects on the newborn have been observed in studies. The neonate’s renal function should be thoroughly assessed when cisplatin is administered in the third trimester, due to its nephrotoxicity. Carboplatin is less nephrotoxic than cisplatin and should be preferred in pregnancy [7]. There are fewer data available on the administration of paclitaxel during pregnancy. The occurrence of placental transfer of paclitaxel in humans is not known. In the second and third trimester, paclitaxel was administered as polytherapy in the majority of cases [7]. The only reported serious adverse event was pyloric stenosis in a fetus treated with paclitaxel, doxorubicin, and cytarabine. The most commonly reported pregnancy complications were oligohydramnios and pre-eclampsia [56].

Pregnancies complicated by maternal cancer are considered high-risk pregnancies and should be managed in a multidisciplinary tertiary center. Prematurity, fetal growth restriction, and fetal loss are more common in these pregnancies than in a normal pregnancy. In our review, four women delivered at term, five women delivered between 34 and 36 weeks of pregnancy, and one woman delivered at 31 weeks of pregnancy. As premature births impair the cognitive and emotional development of children, guidelines recommend aiming for a full-term birth if this is possible [57,58].

A cancer diagnosis during pregnancy can be emotionally challenging for pregnant women and their partners. Feelings of joy, happiness, hope, and anticipation mix with fears, worries, anxieties, and questions [59]. Parents may be concerned about the potential impact of cancer treatment during pregnancy on their child’s health and cognitive, learning, and motor skills, as well as passing on cancer genes to their child. Close monitoring of the newborn is warranted. Women may be afraid that they will not be able to cope with the demands of motherhood and cancer treatment. They may also worry about their own chances of survival [60]. It is important that women are made aware of and counseled on the potential risks when deciding on a treatment option [61]. There are no studies on the impact of cancer diagnosis and treatment during pregnancy on the patient’s partner’s psychological well-being, the quality of the patient’s relationship to their partner, and the couple’s sexual functioning and experiences. The partner should not be excluded from decisions, and psychological support therapy should be offered for patients’ families [61]. To our knowledge, there are still no long-term observational studies of patients’ oncological and obstetric outcomes. In our literature search, the longest follow-up was 42 months after delivery with the normal growth and development of the child and a woman with no signs of disease. The major strength of our article is that we add to the literature a new detailed case of high-grade serous ovarian cancer diagnosed during pregnancy with the longest follow-up of ten years. The limitation of our review is that only studies in English were searched. However, high-grade ovarian cancer during pregnancy is very rare and new important data should not be missed.

In summary, our review has shown that chemotherapy for advanced high-grade ovarian cancer diagnosed during pregnancy is an option to preserve the pregnancy and also to continue the pregnancy until 37 weeks of gestation to ensure the normal growth and development of the baby. Moreover, exploratory laparotomies did not affect the pregnancies. Since most of the cases in our review were older than ten years, laparoscopic or robot-assisted procedures are now an option for staging and diagnosis.

## 5. Conclusions

High-grade serous ovarian cancer diagnosed during pregnancy is extremely rare. Care by a multidisciplinary team including gynecologic oncologists, obstetricians, radiologists, pathologists, anesthesiologists, and neonatologists is necessary. The obstetric and maternal outcomes of high-grade serous ovarian cancer depend on the stage and week of pregnancy when the diagnosis was given. Women must be informed of the possible risks when deciding on a treatment option. As only 11 cases of high-grade serous ovarian cancer in pregnancy were found in the literature and one case was added by us, more case reports and series with longer follow-up periods are needed.

## Figures and Tables

**Table 1:** Patient characteristics.

First Author	Year	Patient Age	Tumor Localization	Histology	FIGO Stage
King [28]	1990	24	Right ovary	HGSC	IIIC
Otton [29]	2001	31	Right ovary	HGSC	IC
Sood [30]	2001	33	Right ovary	HGSC	IIIC
Ferrandina [31]	2005	40	Bilateral ovaries	HGSC	IIIC
Mantovani [32]	2007	34	Right ovary	HGSC	IIIC
Modares Gilani [33]	2007	42	Right ovary	HGSC	IIIC
Rouzi [34]	2009	32	Left ovary	HGSC	IIIC
Smith [35]	2013	36	Left ovary	HGSC	IIB
Vivod	2014	36	Bilateral ovaries	HGSC	IIIC
Xu [36]	2019	34	Bilateral ovaries	HGSC	IIIC
Bacalbasa [37]	2020	27	Right ovary	HGSC	IIIC
Tremblay [38]	2023	40	Bilateral ovaries	HGSC	IIIC

FIGO, The International Federation of Gynecology and Obstetrics; HGSC, high-grade serous cancer.

**Table 2:** Symptoms, gestational age at diagnosis, and work-up.

First Author	Symptoms/Signs	Gestational Age at Diagnosis (Weeks)	Radiologic Work-up	Tumor Maximal Diameter (mm)	Ca-125 at Diagnosis (U/mL)
King [28]	Asymptomatic	9	US	100	62
Otton [29]	Abdominal pain	6	US	80	562
Sood [30]	Abdominal pain	27	US	100	568
Ferrandina [31]	Asymptomatic	14	US	180	1240
Mantovani [32]	Asymptomatic	9	US	51	751
Modares Gilani [33]	Asymptomatic	18	US	80	1000
Rouzi [34]	Abdominal pain	18	US + MR	150	580
Smith [35]	Asymptomatic	9	US	110	183
Vivod	Abdominal pain	28	US + MR	140	801
Xu [36]	Abdominal pain	18	US	150	125
Bacalbasa [37]	Pelvic pain	9	US + MR	40	NS
Tremblay [38]	Asymptomatic	27	US + MR	100	769

MR, magnetic resonance; NS, not specified; US, ultrasound.

**Table 3:** Management of high-grade serous ovarian cancer during pregnancy.

First Author	Week at Surgery	Surgery in Pregnancy	Surgery at Delivery	Chemotherapy during Pregnancy
King [28]	16	Exploratory laparotomy, RSO, left ovarian biopsy, partial omentectomy, small bowel nodule biopsy	No	Six cycles of cisplatin and cyclophosphamid
Otton [29]	16	Exploratory laparotomy, ovarian cystectomy	Hysterectomy, BSO, omentectomy, right pelvic and para-aortic LND, no clinical evidence of macroscopic disease	Four cycles of cisplatin
Sood [30]	28	Exploratory laparotomy, RSO, infracolic omentectomy, optimal cytoreduction	Exploratory laparotomy, hysterectomy, LSO, suboptimal cytoreduction	Three cycles of cisplatin and paclitaxel
Ferrandina [31]	15	Exploratory laparotomy, BSO, infracolic omentectomy, appendectomy, multiple biopsies	Exploratory laparotomy, hysterectomy, multiple biopsies	Six cycles of cisplatin
Mantovani [32]	17	Exploratory laparotomy, RSO, left ovarian biopsy, omental biopsy, removal of two pelvic metastasis	Exploratory laparotomy, hysterectomy, LSO, removal of the superior third of the vagina, omentectomy, pelvic LND, appendectomy, multiple biopsies	Five cycles of paclitaxel
Modares Gilani [33]	20	Exploratory laparotomy RSO, partial omentectomy, lymph node sampling, multiple biopsies	Exploratory laparotomy, hysterectomy, LSO, omentectomy	Four cycles of carboplatin and paclitaxel
Rouzi [34]	20	Exploratory laparotomy LSO, omental biopsy	Exploratory laparotomy, hysterectomy, RSO, omentectomy, multiple biopsies	Four cycles of cisplatin and docetaxel
Smith [35]	12	Exploratory laparotomy, LSO, para-aortic and left pelvic LND, omentectomy, multiple biopsies, appendectomy	No	Four cycles of intraperitoneal carboplatin and IV paclitaxel
Vivod	28	Laparoscopic exploration, multiple peritoneal biopsies	Hysterectomy, BSO, omentectomy, resection of the rectosigmoid colon with anastomosis, pelvic peritonectomy + intraperitoneal chemotherapy (cisplatin)	No
Xu [36]	21	Exploratory laparotomy, BSO, infra-colic omentectomy	Hysterectomy, pelvic and para-aortic LND, omentectomy, appendectomy, partial sigmoidectomy, all apparent independent cancer nodules were resected	Four cycles of cisplatin and docetaxel
Bacalbasa [37]	10	Laparoscopic exploration, cystectomy, no pathological aspect was revealed	At 14 weeks termination of pregnancy, hysterectomy, BSO, omentectomy, pelvic and para-aortic LND, multiple biopsies	No
Tremblay [38]	27	No surgery US-guided tumor biopsy	Hysterectomy, BSO, omentectomy, all apparent independent cancer nodules were resected + HIPEC (cisplatin)	Three cycles of cisplatin and paclitaxel

BSO, bilateral salpingo-oopherectomy; HIPEC, hyperthermic intraperitoneal chemotherapy; IV, intravenous; LND, lymphadenectomy; LSO, left salpingo-oopherectomy; RSO, right salpingo-oopherectomy; US, ultrasound.

**Table 4:** Obstetric and baby outcomes.

First Author	Gestational Age at Delivery (Weeks)	Mode of Delivery	Pregnancy Outcome	Birth Baby Weight (Grams)	Baby Outcome
King [28]	37	Vaginal	Live birth	3060	Normal growth and development at 24 months of age
Otton [29]	31	Cesarean section	Live birth	1740	Normal growth and development at 12 months of age
Sood [30]	37	Cesarean section	Live birth	2800	Normal growth and development at 30 months of age
Ferrandina [31]	36	Cesarean section	Live birth	3000	Normal growth and development at 42 months of age
Mantovani [32]	38	Cesarean section	Live birth	2490	Normal growth and development at 16 months of age
Modares Gilani [33]	35	Cesarean section	Live birth	2600	Normal growth and development at 6 months of age
Rouzi [34]	34	Cesarean section	Live birth	2245	The baby died 5 days after delivery because of multiple congenital anomalies which were diagnosed before starting chemotherapy
Smith [35]	37	Cesarean section	Live birth	2126	Normal growth and development at 7 months of age
Vivod	31	Cesarean section	Live birth	1360	Normal growth and development at 9 years of age
Xu [36]	35	Cesarean section	Live birth	2100	Normal growth and development at 33 months of age
Bacalbasa [37]	Abortion	/	/	/	/
Tremblay [38]	36	Cesarean section	Live birth	2965	Normal growth and development 22 months of age.

**Table 5:** Post-delivery management and maternal outcome.

First Author	Surgery after Pregnancy	Chemotherapy after Pregnancy	Maternal Oncologic Outcome
King [28]	Six weeks postpartum laparotomy, hysterectomy, LSO, omentectomy	Eight courses of cisplatin and etoposide, Intraperitoneal chromic phosphate	Twenty-four months after delivery WSOD
Otton [29]	No	Two cycles of carboplatin and paclitaxel	Twelve months after delivery WSOD
Sood [30]	No	Three cycles of cisplatin and paclitaxel	The patient died of recurrent ovarian cancer 29 months after diagnosis
Ferrandina [31]	Twenty-four months after delivery recurrence of ovarian cancer and removal of nodules in pelvis	Six cycles of carboplatin and paclitaxel 24 months after delivery	Forty-two months after delivery WSOD
Mantovani [32]	No	Six cycles of carboplatin and paclitaxel	Sixteen months after delivery WSOD
Modares Gilani [33]	No	Three cycles of carboplatin	Six months after delivery WSOD
Rouzi [34]	No	Two cycles of cisplatin and docetaxel	Eight months after delivery WSOD
Smith [35]	Twelve weeks after delivery, robotic-assisted hysterectomy, RSO, peritoneal biopsies, para-aortic LND	Two cycles of intraperitoneal carboplatin and IV paclitaxel	Seven months after delivery WSOD
Vivod	No	Six cycles of carboplatin and paclitaxel	Nine years after delivery WSOD
Xu [36]	No	Four cycles of cisplatin and docetaxel for recurrence 24 months after delivery—one cycle of cisplatin and docaxel, six cycles of pegylated liposomal doxorubicin and bevacizumab	Recurrence 24 months after delivery positive pelvic lymph nodes, right supraclavicular lymph node) 33 months after delivery WSOD
Bacalbasa [37]	No	NS	One week after surgery WSOD
Tremblay [38]	No	Three cycles of cisplatin and paclitaxel	Twenty-two months after delivery WSOD

IV, intravenous; LND, lymphadenectomy; LSO, left salpingo-oopherectomy; NS, not specified; RSO, right salpingo-oopherectomy; WSOD, without signs of disease.
